# Unveiling Aging and Alzheimer's Disease–Associated Dynamics of LINE1 DNA Content and Protein Expression in Mouse Brains

**DOI:** 10.1111/acel.70296

**Published:** 2025-11-17

**Authors:** Minyan Jiang, Cheng Zhang, Juanlin Chen, Yanmei Qi, Lina Zhu, Zetong Liu, Jianfei Li, Tao Zhou, Xu Wang, Xihan Guo

**Affiliations:** ^1^ School of Life Sciences Yunnan Normal University Kunming Yunnan China; ^2^ Yeda Institute of Gene and Cell Therapy Taizhou Zhejiang China

**Keywords:** aging, Alzheimer's disease, amyloid‐β, LINE1, sex differences

## Abstract

Despite the long interspersed nuclear element‐1 (LINE1, L1) retrotransposons having been implicated in Alzheimer's disease (AD), a fundamental understanding of the AD‐specific lifespan‐long trajectory of L1 has been limited. Here, we characterize the content and expression of L1 covering four brain regions (hippocampus, prefrontal cortex, cerebellum, and the rest of brain tissue) of APP/PS1 mice, a murine model of AD, and their wild‐type C57BL/6 littermates from 3 to 24 months of age. We report that both L1 content (indicated by DNA copy number) and expression (indicated by protein levels of L1‐encoded ORF1 and ORF2) across brain regions had nonlinear, U‐shaped associations with age in wild‐type and APP/PS1 mice. Compared to age‐matched wild‐types, APP/PS1 mice constantly have significantly decreased L1 content but increased L1 expression, suggesting L1 differences between wild‐type and APP/PS1 mice establish early and remain stable throughout the life course. Strikingly, L1 content and expression in wild‐type and APP/PS1 mice are sexually different, depending on age and brain region. The appearance of L1 alteration precedes the onset of β‐amyloidosis by 3 months in APP/PS1 mice, and β‐amyloidosis is positively correlated with L1 content and expression in males but anti‐correlated with L1 content in females of both wild‐type and APP/PS1 mice. Overall, this study (i) reveals an unanticipated U‐shaped trajectory of L1 content and expression in both normal and pathological aging of mouse brains and (ii) discerns specific changes in L1 content and expression tied to AD neuropathology in a sex‐different manner.

## Graphical abstract

A fundamental understanding of the Alzheimer’s disease (AD) specific trajectory of LINE1 has been limited. This study reveals an unanticipated U‐shaped trajectory of LINE1 content and expression in both wild‐type and AD mouse brains, and discerns specific changes in LINE1 content and expression tied to AD neuropathology in a sex‐different manner. Our data provide a valuable resource for probing normal and pathological aging‐related courses of LINE1 content and expression in mouse brain over the 2 year‐long period of lifespan.

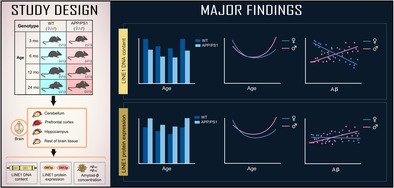

Alzheimer's disease (AD) is the most common cause of dementia and its prevalence is likely to substantially increase in the near future due to population aging (Agirman et al. 2021). Although extracellular deposition of amyloid‐β (Aβ) and intracellular accumulation of hyperphosphorylated Tau are two leading pathological hallmarks, the etiology of AD is complex. New perspectives for understanding AD etiology are therefore vitally important (Guo et al. 2024).

Long interspersed nuclear element‐1 (LINE1, L1) constitutes ~17% of the genome (Figure 1A), a sequence space that vastly eclipses that of the coding genome, thereby making L1 a subject of great research interest (Percharde et al. 2018). Dysregulation of L1‐derived function is detrimental (Xiong et al. 2021). Several studies have described the links between L1 and AD pathogenesis (Feng et al. 2024; Guo et al. 2018; Macciardi et al. 2022; Hanna et al. 2021). Although these works provided snapshots of L1 in AD, they provided limited understanding of the differences in L1 between AD and healthy brains, the sequence of L1 dysregulation and AD‐associated neuropathology, and the potential sex‐dependent effects.

**FIGURE 1:**
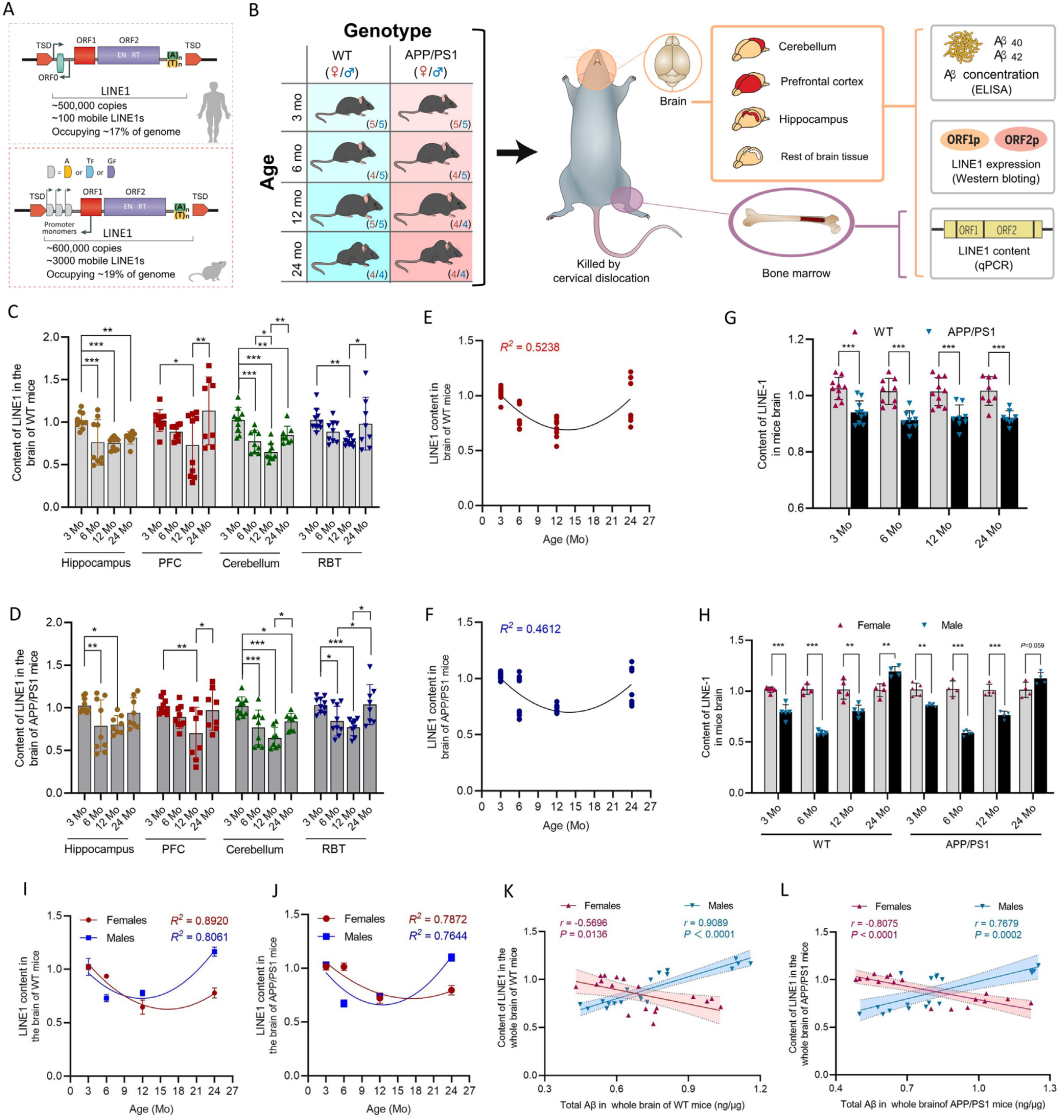
Baseline characteristics of LINE1 (L1) content in WT and APP/PS1 mouse brain. (A) The structure of L1 elements in humans (up) and mouse (down). Adapted from Faulkner and Garcia‐Perez (2017). Abbreviations: EN, endonuclease; RT, reverse transcriptase; TSD, target‐site duplication. (B) Schematic workflow of this study. The number of samples of two sexes analyzed in each group is listed. (C, D) Bar plots showing L1 content in hippocampus, prefrontal cortex (PFC), cerebellum, and the rest of brain tissue (RBT) in WT (C) and APP/PS1 (D) mice from 3 to 24 months of age. LINE1 content was measured with GAPDH as internal control (see Supporting Information: Methods). LINE1 content at 3 months was calculated as 1. (E, F) Plots showing a U‐shaped trajectory of L1 content with age in WT (E) and APP/PS1 (F) mice. Nonlinear curve provides best fit with *R* ^*2*^ = 0.5238 and 0.4612 for WT and APP/PS1 mice, respectively. L1 content at 3 months was calculated as 1. (G) Bar plots showing L1 content in whole brain of WT versus APP/PS1 mice at different age points. L1 content of females at indicated ages was calculated as 1. (H) Bar plots showing age‐dependent sex differences in LINE1 content in whole brain of WT and APP/PS1 mice. (I, J) Plots showing the age trajectory of LINE1 content in two sexes of WT (I) and APP/PS1 (J) mice. In WT mice (I), nonlinear curve provides best fit with *R* ^*2*^ = 0.8920 and 0.8061 for females and males, respectively. In APP/PS1 mice (J), nonlinear curve provides best fit with *R* ^*2*^ = 0.7872 and 0.7644 for females and males, respectively. L1 content at 3 months of each sex was calculated as 1. (K, L) Integrated analysis of the relationships between L1 content and Aβ concentration in whole brain of WT (K) and APP/PS1 mice (L) highlights sex‐divergent associations. The shaded area shows a linear fit ±95% confidence interval. *r* is the Pearson's correlation coefficient. *p* value was calculated using two‐sided Pearson's correlation test. Data are presented as mean ± SD of at least three biological replicates for all experiments. Each point in C–H, K, and L represents one animal. Statistical significance was determined by one‐way ANOVA with Tukey's post hoc tests for C and D, and Student's *t*‐test for G and H. *p < 0.05, **p < 0.01, ***p < 0.001.

The mobile nature of L1 and the gradual onset of AD mean that longitudinal assessments to track L1 trajectories may offer a more comprehensive insight into the association between L1 and AD. Here, we characterize the content and expression of L1 and elucidate their correlations with Aβ in both sexes of wild‐type (WT) and APP/PS1 (AD) mouse brains. To grasp the spatiotemporal course of L1, we conduct experiments in mice aged from 3 to 24 months covering four brain regions (Figure 1B).

L1 DNA was quantified in four brain regions. The results showed that L1 content in each brain region showed nonlinear associations with age in WT and AD mice (Figure 1C,D; Figure S1A–H). When brain regions were aggregated, U‐shaped associations between L1 content and age were observed, with a decrease of close to 30% in WT and AD mice between 3 and 12 months (Figure 1E,F). Moreover, we compared data between age‐matched WT and AD mice. The results showed that, at each age point, L1 content in AD mice was significantly lower than that in WT mice, although the significance was less prominent in some target regions of L1 (Figure 1G; Figure S1I–L).

Our further explorations delved into sex differences in L1 content. We found that, in each brain region, L1 content exhibited sex differences in either WT or AD mice (Figure S2A–D). When considering all brain regions together, L1 content in WT and AD mice was decreased in males when compared to females at 3–12 months but was dominated by males at 24 months (Figure 1H). These results highlight the importance of taking age into account when studying sex differences in L1. In each brain region, the age course of L1 content was different between males and females (Figure S2E–H). When brain regions were aggregated, both sexes of WT and AD mice displayed U‐shaped associations between L1 content and age (Figure 1I,J).

We next turned our attention to ask whether L1 content is related to Aβ. We first quantified Aβ_40_ and Aβ_42_ concentrations in WT and AD mice (Figure S3A–D). When compared with WT mice, AD mice had significantly increased levels of Aβ in some brain regions since 6 months of age (Figure S3E–H). When pooling all brain regions, L1 content and Aβ were positively correlated in males but were negatively correlated in females in WT and AD mice (Figure 1K,L).

We next asked whether the U‐shaped pattern of L1 content is specific to brain tissues. The results showed that the association of L1 content with age is discordant among L1 subfamilies in the bone marrow of WT or AD mice (Figure S4A,B). In addition, unlike that observed in the brain, L1 content in the bone marrow was indistinguishable between WT and AD mice (Figure S4C–F). In WT and AD mice, the association of L1 content with age was discordant between the two sexes (Figure S4G), indicating the possibility of a sex‐specific regulatory mechanism of L1 in the bone marrow. L1 content in males was lower than that in females in either WT or AD mice at 6, 12, and 24 months and the gap between females and males tended to widen as age increased (Figure S4H).

Furthermore, we found that the age course of ORF1p and ORF2p expression varies across brain regions in both WT and AD mice (Figure 2A,B; Figure S5A–H). When pooling all brain regions, ORF1p and ORF2p were positively correlated (Figure S5I) and showed nonlinear, U‐shaped associations with age in both WT and AD mice (Figure 2C,D). While ORF1p and ORF2p expression in AD mice was significantly higher than in age‐matched WT mice (Figure 2E,F), the ratio of ORF2p to ORF1p was unchanged between them (Figure S5J). Interestingly, L1 content and expression were concordant in both WT and AD mice (Figure 2G).

**FIGURE 2:**
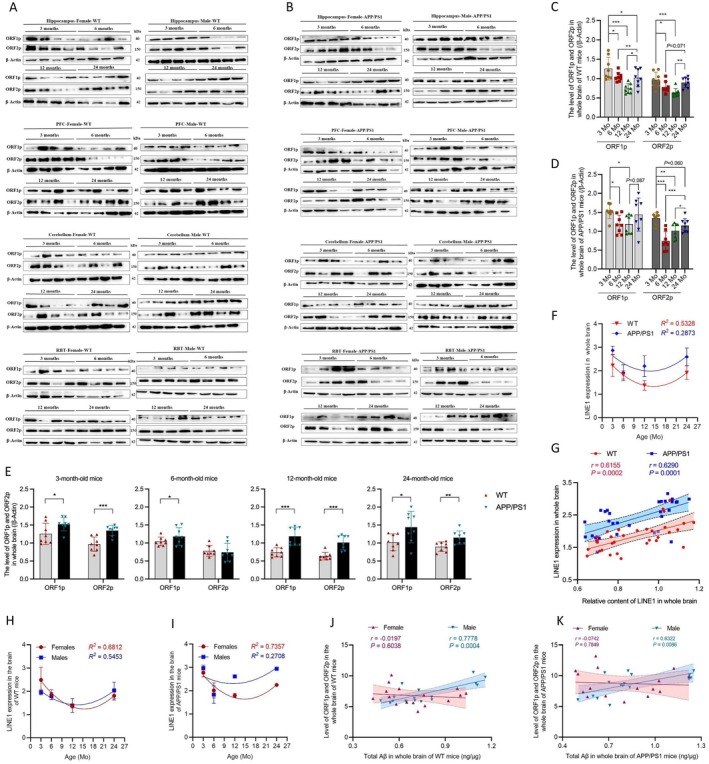
Baseline characteristics of LINE1 (L1) expression in WT and APP/PS1 mouse brains. (A, B) Immunoblotting of hippocampus, prefrontal cortex (PFC), cerebellum, and the rest of brain tissue (RBT) lysates from WT (A) and APP/PS1 (B) mice at 3–24 months of age for ORF1p and ORF2p, with β‐actin as a loading control. (C, D) Bar plots showing ORF1p and ORF2p levels in the whole‐brain of WT (C) and APP/PS1 (D) mice from 3 to 24 months of age. (E) Bar plots showing ORF1p and ORF2p levels in whole brain of WT versus APP/PS1 mice at different age points. (F) Age trajectory of L1 expression (sum of ORF1p and ORF2p) in WT and APP/PS1 mice. Nonlinear curve provides best fit with *R* ^*2*^ = 0.5328 and 0.2873 for WT and APP/PS1 mice, respectively. (G) Positive correlation between L1 content and expression in WT and APP/PS1 mice. (H, I) Plots showing age‐dependent sex difference in LINE1 expression in WT (H) and APP/PS1 (I) mice. In WT mice (H), nonlinear curve provides best fit with *R* ^*2*^ = 0.6812 and 0.5453 for females and males, respectively. In APP/PS1 mice (I), nonlinear curve provides best fit with *R* ^*2*^ = 0.7357 and 0.2708 for females and males, respectively. (J, K) Integrated analysis of the relationships between L1 expression and Aβ concentration in whole brain of WT (J) and APP/PS1 mice (K) highlights sex‐divergent associations. In G, J, and K, the shaded shows a linear fit ±95% confidence interval *r* is the Pearson's correlation coefficient. *p* value was calculated using two‐sided Pearson's correlation test. Data are presented as mean ± SD of at least three biological replicates for all experiments. Each point in C–E, G, J, and K represents one animal. Statistical significance was determined by one‐way ANOVA with Tukey's post hoc tests for C and D and Student's *t*‐test for E. *p < 0.05, **p < 0.01, ***p < 0.001.

Moreover, ORF1p and ORF2p expression was sex‐specific (Figure S5K–N). Considering all brain regions together, we found that the sex difference in L1 expression was age dependent (Figure 2H,I; Figure S5O). Moreover, L1 expression was positively correlated with Aβ level in males of WT and AD mice. This relationship, however, was not observed in females of either WT or AD mice (Figure 2J,K).

Taken together, we have conducted a comprehensive analysis of L1 content and expression across a 2‐year lifespan in both sexes of WT and AD mice. There are three findings that make this study noteworthy. First, we find L1 content and expression display U‐shaped associations with age in WT and AD mouse brain. To our knowledge, this age‐associated trend has not been reported before in mice. While the mechanisms shaping the U‐shaped curve are unclear, we speculate these may involve age‐related changes in brain such as cell type composition changes, reactive gliosis, or selection bias caused by cell death. In agreement, the expression of retrotransposons in the head of *Drosophila* also follows a U‐shaped curve, with a trend toward reduction in middle‐aged adults and reactivation in older individuals (Giordani et al. 2022). Moreover, ORF1p and ORF2p levels in the cortex of WT mice decrease stepwise from 3 to 24 months (Floreani et al. 2022). In the current study, L1 content and protein expression remained unchanged between 3 and 24 months. In line with it, L1 content and RNA expression in mouse livers remained unchanged between 5 and 24 months, and a strong increase was observed until 36 months (De Cecco et al. 2013). All these data support a nonlinear association between L1 and age. Coupled with these data, we suggest there may be a J‐shaped association of L1 content and expression with advanced age.

Second, LINE1 differences between WT and AD mice establish early and remain stable throughout the life course. While L1 content and expression are concordant in both WT and AD mice, L1 content is repressed but L1 expression is de‐repressed throughout the lifespan of AD mice when compared to WT mice, supporting a growing body of literature describing L1 dysregulation as involved in AD. The discordance in L1 content and expression has been observed in the amygdala of juvenile rats, where the males have decreased DNA content but increased mRNA expression as compared to females (Cuarenta et al. 2021). The decreased content but increased expression of L1 in AD mice raises the possibility that ORF1p and ORF2p may carry out roles nonrelevant to L1 retrotransposition in AD. For example, nonautonomous retrotransposons such as Alu and SINE‐VNTR‐Alu may mobilize in *trans* by hijacking L1‐encoded proteins (Fukuda et al. 2021; Raiz et al. 2012). Another possibility is that ORF1p and ORF2p, although highly expressed, are less active in driving L1 retrotransposition in AD mice. Indeed, hundreds of sequence‐diverse ORF2 mRNA variants are found in the AD brain, and some of them lack functional reverse transcriptase activity (Nicodemus et al. 2025).

Third, there are age‐dependent sex differences in L1 content and expression in both WT and AD mice. Although the brain is well characterized as a sexually dimorphic organ, the sex difference in brain L1 is a relatively unexplored topic. There is a region‐dependent sex difference in L1 content and mRNA expression in the brain of juvenile rats (Cuarenta et al. 2021). L1 mRNA expression in mouse organs exhibits longitudinal changes in a sex‐specific manner (Stow et al. 2021). Early life stress can mobilize L1 in a sex‐ and region‐specific manner in rats (Cuarenta et al. 2020). We acknowledge that factors contributing to sex‐specific L1 content and expression remain to be further elucidated. One related factor is sex chromosome difference. Expression of transposable elements is associated with the presence and number of Y chromosomes in humans (Teoli et al. 2025). Given that the Y chromosome tends to be lost in mammalian somatic cells with age in mice (unpublished data), the loss of the Y chromosome (Guo and Dai 2025; Guo et al. 2020) may explain why the sex difference of L1 content and expression changed with age. In addition, we find L1 and total Aβ are positively correlated in males but less or anti‐correlated in females. Engineering L1 in the mouse brain to directly test the sexually different roles of L1 in β‐amyloidosis would be valuable. A recent study demonstrated that transcriptional activation of L1 drives changes in microglial morphology and cytokine secretion and impairs the phagocytosis of Aβ (Roy et al. 2024).

In summary, our study provides a valuable resource for probing age‐ and AD‐related courses of L1 content and expression in mouse brain and answers the question of how L1 content and expression differ between WT and AD mice over the 2‐year‐long period of lifespan. Although our study lacks sufficient mechanistic resolution, our findings may guide future studies that will dissect the sex‐specific role of L1 dysfunction in the onset and progression of brain aging under normal and pathological conditions.

## Author Contributions

**Minyan Jiang:** methodology, data curation, validation, and writing – original draft. **Cheng Zhang:** data curation. **Juanlin Chen:** data curation. **Yanmei Qi:** data curation. **Lina Zhu:** data curation. **Zetong Liu:** data curation. **Jianfei Li:** data curation. **Tao Zhou:** methodology. **Xu Wang:** conception, resource, and supervision. **Xihan Guo:** conception, methodology, data curation, validation, writing – review and editing, funding acquisition, and supervision. All authors contributed to the revision and review of this article.

## Disclosure

The authors declare that they have no known competing financial interests or personal relationships that could have appeared to influence the work reported in this paper. The authors declare no Artificial Intelligence Generated Content tools and others based on large language models were used in developing any portion of the manuscript.

## Conflicts of Interest

The authors declare no conflicts of interest.

## Supporting information


**Figures S1–S5.** acel70296‐sup‐0001‐FiguresS1‐S5.docx.

**Appendix S1.** acel70296‐sup‐0002‐AppendixS1.docx.

## Data Availability

The data that support the findings of this study are available on request from the corresponding author. The data are not publicly available due to privacy or ethical restrictions.
