# Supplementary material for: Unveiling Aging and Alzheimer's Disease–Associated Dynamics of LINE1 DNA Content and Protein Expression in Mouse Brains
Source: Aging Cell. 2025 Nov 17;24(12):e70296. doi: 10.1111/acel.70296 (PMC12686561; doi:10.1111/acel.70296)
Supplement: Supplementary file 1 — Figures S1–S5. acel70296‐sup‐0001‐FiguresS1‐S5.docx. [file ACEL-24-e70296-s002.doc]

**Supporting information**

**Unveiling Aging and Alzheimer's Disease–Associated Dynamics of**

**LINE1 DNA Content and Protein Expression in Mouse Brains**

Minyan Jiang^1^, Cheng Zhang^1, a^, Juanlin Chen^1^, Yanmei Qi^1, a^, Lina Zhu^1^, Zetong Liu^1^, Jianfei Li^1^, Tao Zhou^1^, Xu Wang^1,2^, Xihan Guo^1^#

^1^ School of Life Sciences, Yunnan Normal University, Kunming 650500, Yunnan, China

^2^ Yeda Institute of Gene and Cell Therapy, Taizhou, Zhejiang, China

^a^ Present address: Kunming Institute of Zoology, Chinese Academy of Sciences, Kunming, 650223, Yunnan, China

Corresponding author

# Xihan Guo

[guo_xihan@163.com](mailto:guo_xihan@163.com)

[**https://doi.org/10.1111/acel.70296**](https://doi.org/10.1111/acel.70296)

**Note: In the format provided by the authors and unedited**

**Supplemental Figures**


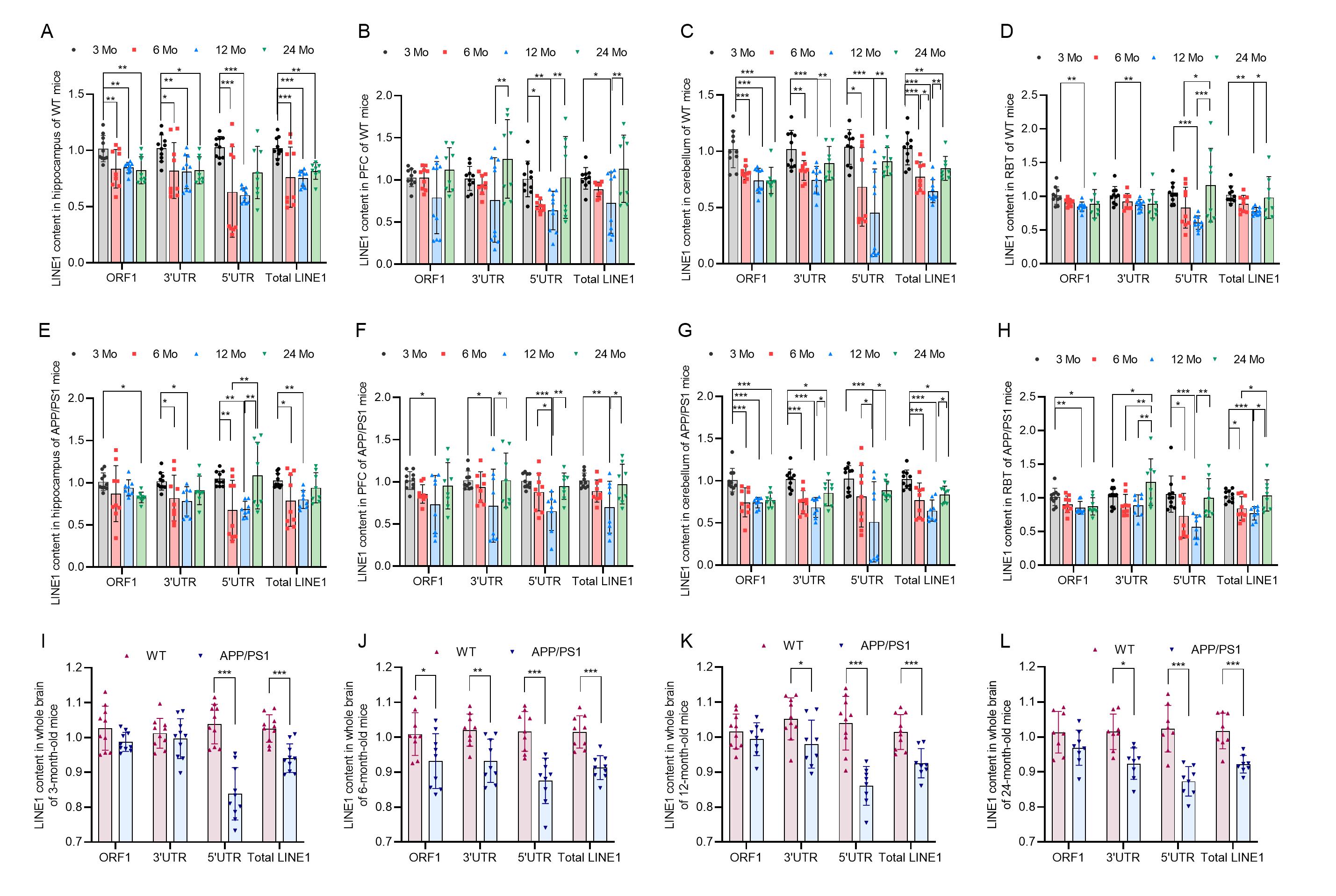


**Supplementary Figure 1**

**Assessment of LINE1 (L1) content across brain regions of WT and APP/PS1 mice.** **(A-H)** Bar plots showing L1 content in hippocampus, prefrontal cortex (PFC), cerebellum, and the rest of brain tissue (RBT) in WT (A-D) and APP/PS1 (E-H) mice from 3 to 24 month-of-age. L1 content was quantified by quantitative PCR (qPCR) with three pairs of primer targeting the ORF1 (open reading frame 1), 3’-UTR (untranslated region) and 5’-UTR, and the total L1 was the mean of these target regions. These amplicons are common to all L1 families. Data was normalized to GAPDH as an internal control. Data were additionally normalized to the 3-month value for each L1 (shown as 1.0) **(I-L)** Bar plots showing L1 content aggregated from four brain regions of mice at 3 (I), 6 (J), 12 (K), 24 month-of-age (L). Data are presented as mean ± SD (standard deviation). At least three biological replicates for all experiments. Each point in A-L represents one animal. Statistical significance was determined by one-way ANOVA with Tukey's post hoc tests for longitudinal data (A-J) and Student's t-test for group comparisons (K-N): **P* < 0.05, ***P* < 0.01, ****P* < 0.001.


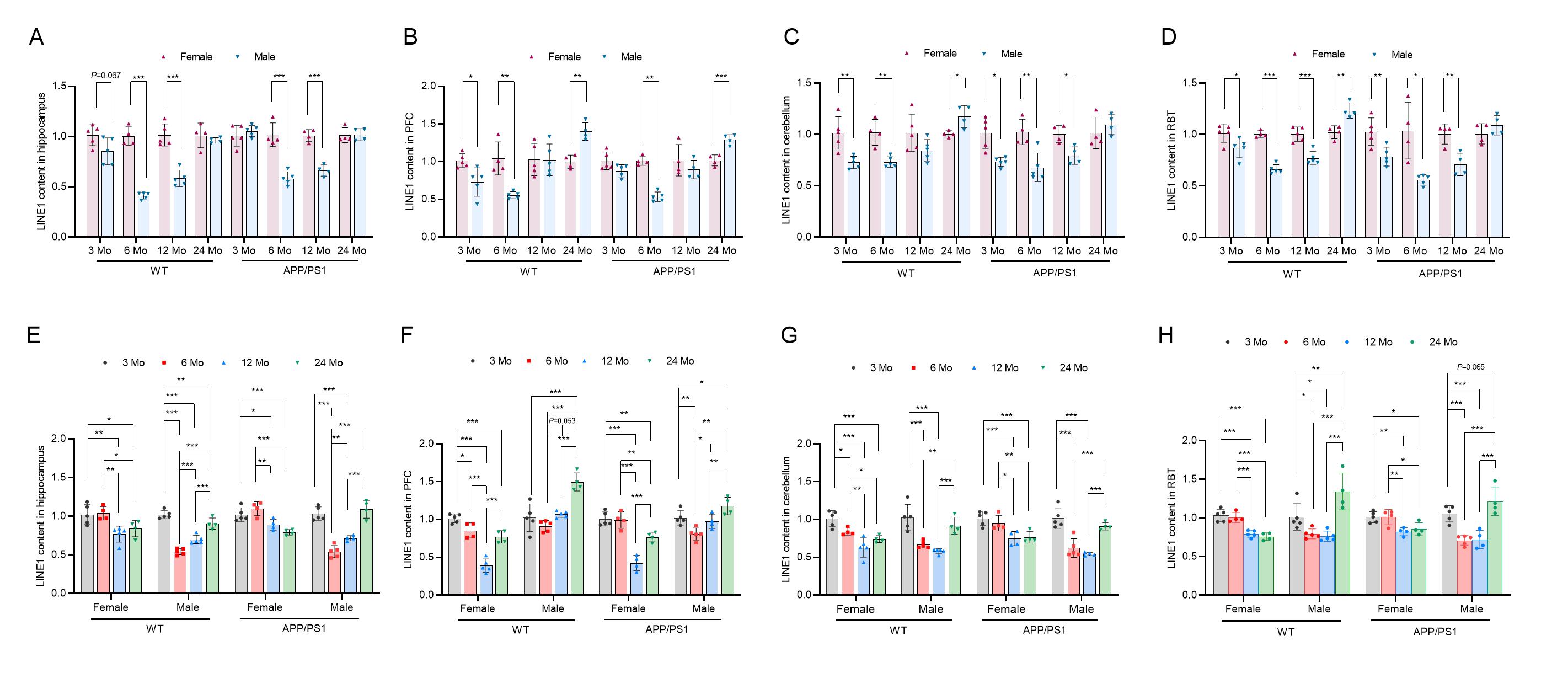
**Supplementary Figure 2**

**Sex difference in LINE1 (L1) content across brain regions of WT and APP/PS1 mice.** **(A-D)** Bar plots showing sex differences in L1 content in hippocampus, prefrontal cortex (PFC), cerebellum, and the rest of brain tissue (RBT) of WT and APP/PS1 mice. **(E-H)** Bar plots showing sex differences in age-related changes of L1 content in the hippocampus, PFC, cerebellum, and RBT of WT and APP/PS1 mice. Data are presented as mean ± SD (standard deviation). At least three biological replicates for all experiments. Each point in A-H represents one animal. Statistical significance was determined by Student's t-test for group comparisons (A-D) and one-way ANOVA with Tukey's post hoc tests for longitudinal data (E-H) and: **P* < 0.05, ***P* < 0.01, ****P* < 0.001.


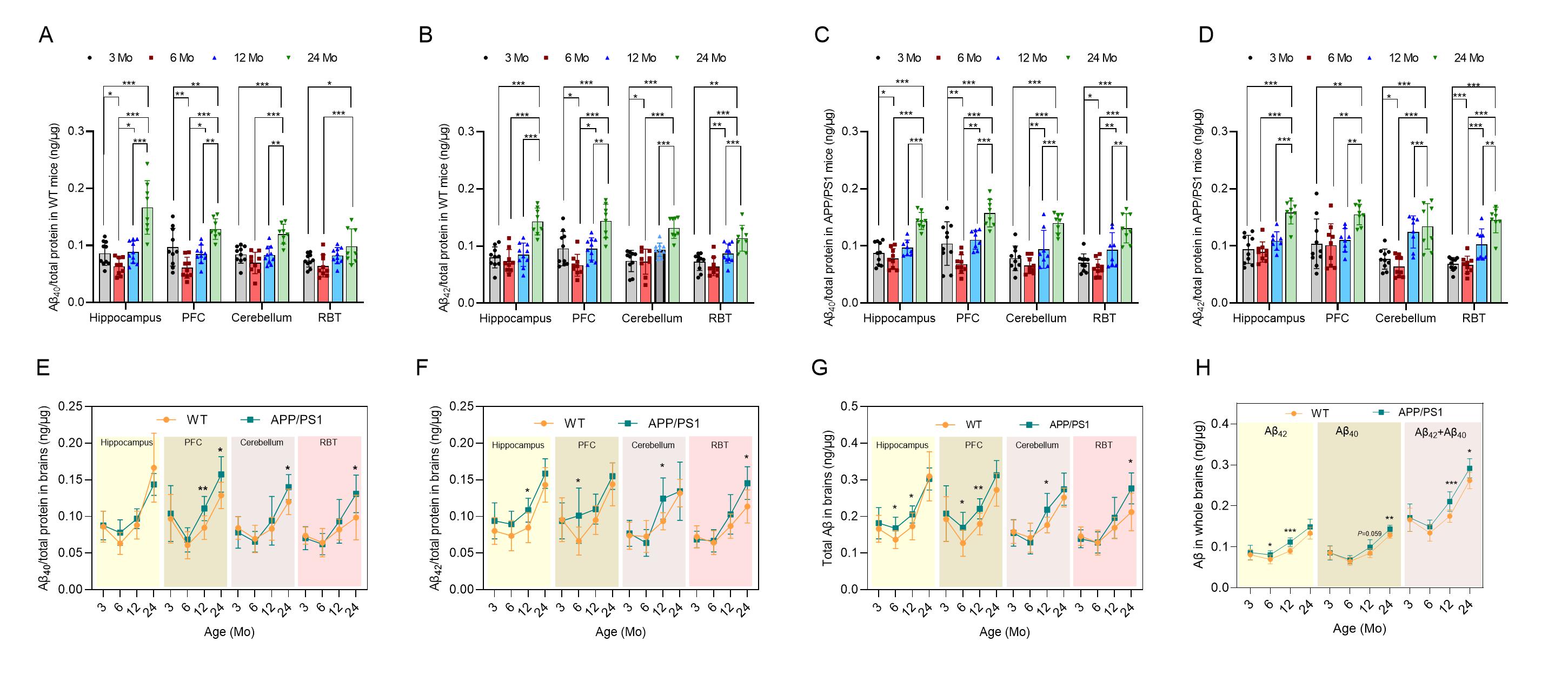


**Supplementary Figure 3**

**Assessment of** **Aβ concentration across brain regions of WT and APP/PS1 mice. (A-D)** Bar plots showing the concentrations of Aβ_40_ (A, C) and Aβ_42_ (B, D) in hippocampus, prefrontal cortex (PFC), cerebellum, and the rest of brain tissue (RBT) in WT (A, B) and APP/PS1 (C, D) mice from 3 to 24 month-of-age. **(E-G)** A comparison of Aβ concentration across brain regions in age-matched WT and APP/PS1 mice. **(H)** A comparison of Aβ concentration in whole brain of age-matched WT and APP/PS1 mice. Data are presented as mean ± SD (standard deviation). At least three biological replicates for all experiments. Each point in A-D represents one animal. Statistical significance was determined by one-way ANOVA with Tukey's post hoc tests for longitudinal data (A-D) and Student's t-test for group comparisons (E-H): **P* < 0.05, ***P* < 0.01, ****P* < 0.001.

**
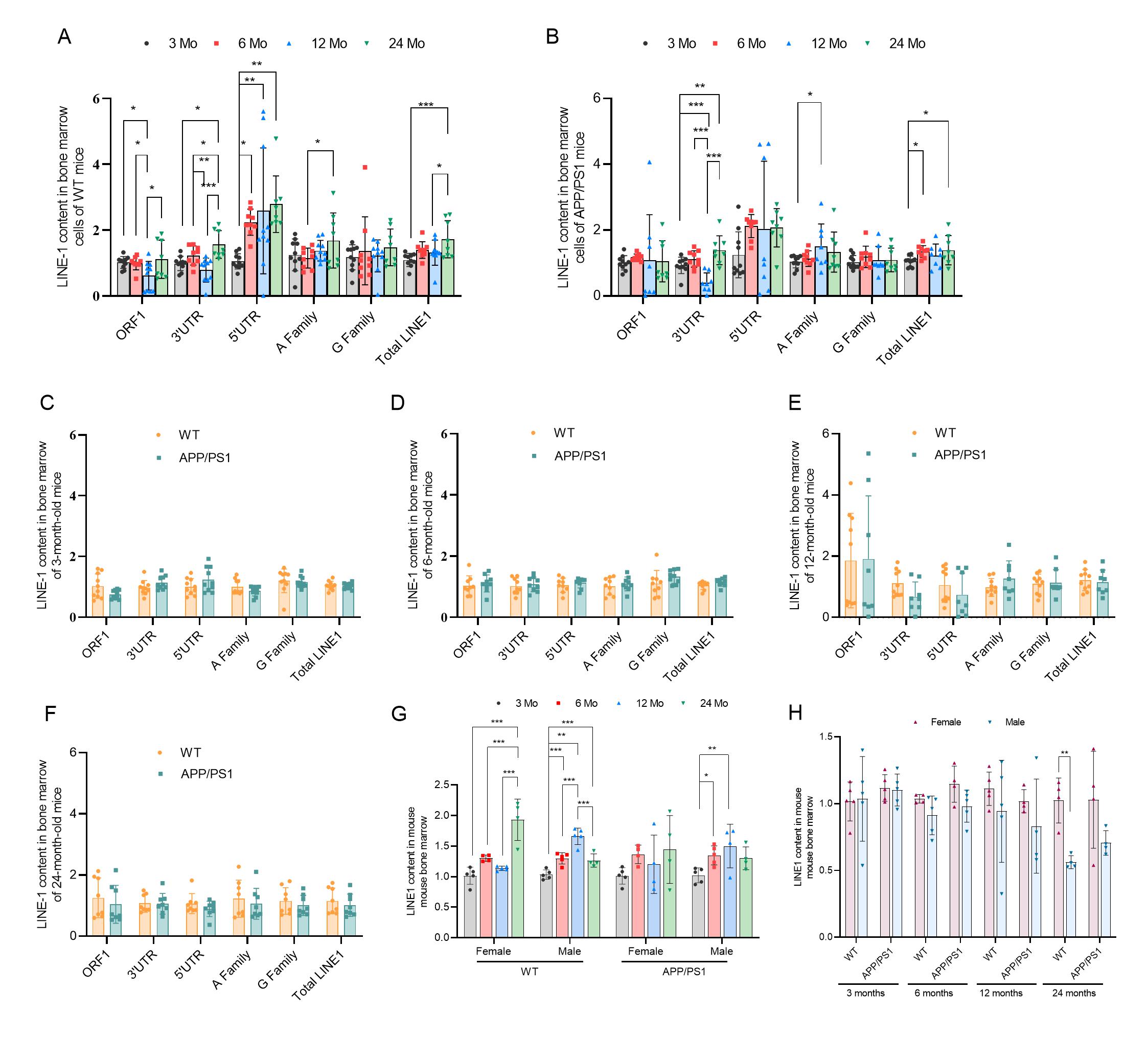
**

**Supplementary Figure 4**

**Assessment of LINE1 (L1) content in bone marrow of WT and APP/PS1 mice.** (A-B) Bar plots showing the quantification of total L1 content in bone marrow of WT (A) and APP/PS1 (B) mice from 4 to 24 months of age. Besides three amplicons common to all L1 families, two amplicons specific to the retrotransposition-competent families (A and G families) were also included. (C-F) Bar plots showing the comparison of L1 content in bone marrow of WT and APP/PS1 mice from 3 to 24 months of age. (G) Bar plots showing sex differences in age-related trajectories of L1 content in WT and APP/PS1 mice. (H) Bar plots showing sex differences in L1 content in bone marrow of age-matched WT and APP/PS1 mice. Data are presented as mean ± SD (standard deviation). At least three biological replicates for all experiments. Each point in A-H represents one animal. Statistical significance was determined by one-way ANOVA with Tukey's post hoc tests for longitudinal data (A, B, G) and Student’s t-test for group comparisons (C-F, H): **P* < 0.05, ***P* < 0.01, ****P* < 0.001.

**
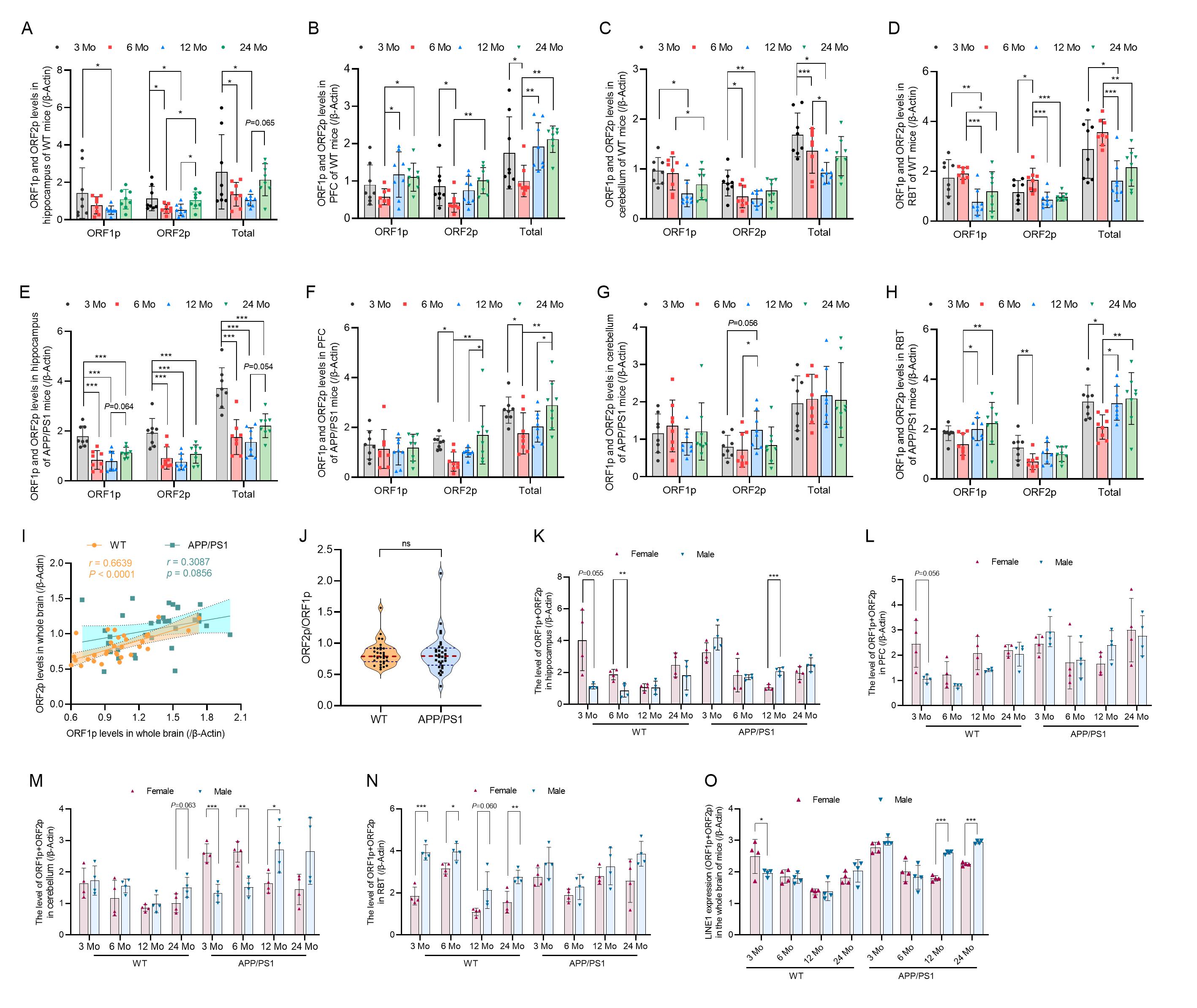
**

**Supplementary Figure 5**

**Assessment of LINE1 (L1) expression across brain regions of WT and APP/PS1 mice.** **(A-H)** Bar plots showing the expression of in ORF1p and ORF2p, in the hippocampus, prefrontal cortex (PFC), cerebellum, and the rest of brain tissue (RBT) in WT (A-D) and APP/PS1 (E-H) mice from 3 to 24 months of age. (I) Pearson’s correlation between ORF1p and ORF2p levels in whole brain of WT and APP/PS1 mice. The shaded area shows a linear fit ± 95% confidence interval. *r* is the Pearson’s correlation coefficient. *P* value was calculated using two-sided Pearson’s correlation test. (J) A violin plot of ORF2p/ORF1p ratio in whole brain of WT and APP/PS1 mice. ns, not significant. Violin plot shows median (center line), 75th and 25th interquartile (top and down lines) (K-N) Bar plots showing sex differences in L1 expression in hippocampus, PFC, cerebellum, and RBT of WT and APP/PS1 mice. (O) Bar plots showing sex differences in L1 expression in whole brain of female and male WT and APP/PS1 mice. Data are presented as mean ± SD (standard deviation). At least three biological replicates for all experiments. Each point in A-O represents one animal. Statistical significance was determined by one-way ANOVA with Tukey's post hoc tests for longitudinal data (A-H) and Student’s t-test for group comparisons (J-O): **P* < 0.05, ***P* < 0.01, ****P* < 0.001.
