# Supplementary material for: Unveiling Aging and Alzheimer's Disease–Associated Dynamics of LINE1 DNA Content and Protein Expression in Mouse Brains
Source: Aging Cell. 2025 Nov 17;24(12):e70296. doi: 10.1111/acel.70296 (PMC12686561; doi:10.1111/acel.70296)
Supplement: Supplementary file 2 — Appendix S1. acel70296‐sup‐0002‐AppendixS1.docx. [file ACEL-24-e70296-s001.doc]

**Supporting information**

**Unveiling Aging and Alzheimer's Disease–Associated Dynamics of**

**LINE1 DNA Content and Protein Expression in Mouse Brains**

Minyan Jiang^1^, Cheng Zhang^1, a^, Juanlin Chen^1^, Yanmei Qi^1, a^, Lina Zhu^1^, Zetong Liu^1^, Jianfei Li^1^, Tao Zhou^1^, Xu Wang^1,2^, Xihan Guo^1^#

^1^ School of Life Sciences, Yunnan Normal University, Kunming 650500, Yunnan, China

^2^ Yeda Institute of Gene and Cell Therapy, Taizhou, Zhejiang, China

^a^ Present address: Kunming Institute of Zoology, Chinese Academy of Sciences, Kunming, 650223, Yunnan, China

Corresponding author

# Xihan Guo

[guo_xihan@163.com](mailto:guo_xihan@163.com)

[**https://doi.org/10.1111/acel.70296**](https://doi.org/10.1111/acel.70296)

Note: In the format provided by the authors and unedited

**Supplemental Materials and Methods**

**1 Animal models and genotyping**

C57BL/6 mice are one of the most widely used inbred strains, commonly serving as a foundational model for transgenic research and are widely used. Transgenic mice were generated via co-injecting vectors for mutant *APP* (Mo/Hu*APP*695^swe^, humanized with Swedish mutation) and mutant *PS1* (lacking exon 9, modeling AD-linked *PS1* mutations), hereafter referred to as APP/PS1 mice. Compared to single-transgenic counterparts, APP/PS1 mice exhibit accelerated Aβ deposition, synaptic degeneration, memory impairment, and neuronal loss, rendering them a robust model for investigation the pathogenesis of AD . Age equivalents between mouse and human are as follows: mice at 3−6, 10−14 and 18−24 months are estimated to be the human lifespan equivalent of approximately 20−30, 38−47, 56−69 years, respectively .

We selected male and female WT and APP/PS1 mice at ages 3, 6, 12, and 24 months (n = 4−5 per group). All mice were maintained in specific pathogen-free (SPF) conditions at 24−26°C and 50% humidity, with a 12 h light/dark cycle and ad libitum access to water and food (provided by Beijing Kela XieLi Feed Co., Ltd.). WT mice were purchased from Kunming Medical University, and APP/PS1 doubly transgenic mice were provided by Professor Keming Zhu and Hongliang Li from Yunnan University. All procedures were approved by the Medical Research Ethics Committee of Yunnan Normal University.

At the second postnatal week, genomic DNA was extracted from tail biopsies (1-2 mm). Polymerase chain reaction (PCR) amplification was performed using primer sequences listed in Table [1](#table1). PCR cycling conditions were as follows: Denaturation at 94 °C for 10 minutes, followed by 40 cycles of denaturation at 94 °C for 5 seconds, annealing at 58 °C for 15 seconds, and extension at 72 °C for 40 seconds, with a final extension at 72 °C for 3 minutes. Electrophoresis results showing three bands corresponding to human *APP* and *PSEN1* and murine *β-Actin* were indicative of APP/PS1 mice, while those showing only the murine *β-Actin* band were identified as WT mice. Genotypes were reconfirmed prior to experiments.

**Table 1.** Primer sequence list

| Primer | Forward (5’→3’) | Reverse (5’→3’) | References |
| --- | --- | --- | --- |
| *β-Actin* | GCTACAGCTTCACCACCACAG | GGTCTTTACGGATGTCAACGTC |  |
| *PS1* | AATAGAGAACGGCAGGAGCA | GCCATGAGGGCACTAATCAT |  |
| *APP* | GACTGACCACTCGACCAGGTTCTG | CTTGTAAGTTGGATTCTCATATCCG |  |
| *GAPDH* | GGAGCGAGACCCCACTAACA | ACATACTCAGCACCGGCCTC |  |
| *ORF1* | ATGGCGAAAGGCAAACGTAAG | ATTTTCGGTTGTGTTGGGGTG |  |
| *3’UTR* | AGACTGCCATAGCCAGGGATC | CAGCTACATCTGCGTCCTTTCA |  |
| *5’UTR* | CAGCCGGCCACCTTCC | GGTCCCGGACCAAGATGG |  |
| *A-Family* | TGAGCACTGAAACTCAGAGGAG | GATTGTTCTTCTGGTGATTCTGTTA |  |
| *G-Family* | TGCCCACTGAAACTAAGGAGA | GCTTGTTCTTCAGGTGACTCTGT |  |

**2 Tissue preparations**

Following cervical dislocation, craniotomy was performed using ophthalmic forceps and microdissection scissors. Brains were extracted, rinsed in PBS, and dissected to isolate the hippocampus, PFC, cerebellum, and RBT. Samples were flash-frozen in liquid nitrogen and stored at −80°C. Samples were flash-frozen in liquid nitrogen and stored at −80°C for subsequent molecular sample extraction.

**3 Aβ quantification by ELISA**

Each brain tissue sample was homogenized in RIPA lysis buffer containing protease inhibitors using sonication, and then incubated on ice for 30 minutes with intermittent vortexing every 10 minutes. After centrifugation at 4 °C for 15 minutes, the supernatant was collected. Total protein concentration was determined by measuring absorbance (OD) values using a bicinchoninic acid (BCA) assay kit (GLPBIO, USA). Following the instructions of the mouse Aβ enzyme-linked immunosorbent assay (ELISA) kit (Jiangsu Meibiao Biotechnology, China), the OD values for Aβ_40_ and Aβ_42_ were measured and their absolute concentrations calculated. Finally, the relative expression level of Aβ_40_ and Aβ_42_ were normalized to the total protein concentration.

**4 Investigating LINE1 (L1) content by quantitative PCR (qPCR)**

L1 DNA was quantified using real-time qPCR from cells of four brain regions. Since many L1 insertions fail to incorporate the full-length element, but rather are 5’ truncated , we used three pairs of primers were targeted to different regions of L1 body (ORF1, 3'-UTR, 5'-UTR) and the total L1 was summed from these regions. L1 DNA was normalized to GAPDH and HERV-H (human endogenous retrovirus H) as internal controls that are not subject to cell-to-cell variation. After testing for specificity and efficiency, these primers produced comparable result in identical samples normalized using GAPDH and HERVH. Considering whether HERVH in mice brain is transcribed upon aging or AD pathogenesis remains unknown, GAPDH was used as an internal control for further experiments.

DNA was extracted from four parts of the brain according to the manufacturer's instructions. Bone marrow cells were collected by perfusing the femur with PBS. The experimental system was prepared using the SYBR Green qPCR Master Mix (GLPBIO, USA), with primer sequences referenced from other research teams as detailed in Table [1](#table1). The RT-qPCR cycling conditions were as follows: 95 °C for 5 minutes to activate the DNA polymerase, followed by 95 °C for 30 seconds, 55 °C for 30 seconds, and 72 °C for 1 minute for a total of 40 cycles, and finally, a melting curve analysis was performed (95 °C for 15 seconds, 60 °C for 60 seconds, and 95 °C for 15 seconds for one cycle). The qPCR reactions were carried out on a StepOne Plus instrument (Applied Biosystems, USA). All samples were run in parallel, and a minimum of 3 independent experiments were performed. The 2^-ΔΔCt^ method was used to calculate the relative copy number of L1 (normalized to GAPDH).

**5 Detecting protein expression of L1 via Western blotting (WB)**

L1 expression can be measured at several points in its life cycle, including L1 RNA expression and L1-encoded ORF1p and/or ORF2p expression, and somatic insertion. Previous study has validated that the protein quantification may be a simpler and more robust measure of L1 expression than RNA . In addition, the majority of L1 RNAs in cells are nonfunctional for retrotransposition, but rather are transcripts that variously consist of a short-lived, pre-spliced mRNA; along noncoding RNA; or a persistent structural RNA . Thus, we used WB to measure the expression of ORF1p and ORF2p in different brain regions of WT and APP/PS1 mice.

Total proteins were extracted from different brain regions, and the protein concentration was measured with the BCA kit (GLPBIO, USA) to ensure an equal loading of samples between lanes. Protein samples (15−30 μg per well) were separated by SDS-PAGE electrophoresis. Following electrophoresis, a "sandwich model" was constructed with the order of "sponge-filter paper-gel-PVDF membrane-filter paper-sponge" to transfer proteins onto the PVDF membrane using the principle of charge transfer. Based on the size of the target band, a strip was cut at the corresponding position on the protein marker. The membrane was then placed in a 5% defatted milk solution and blocked for 2 hours at room temperature on a shaker.

Primary antibodies working solutions were prepared and diluted in TBST as the following concentrations: β-Actin (1:2000; Boster, China), ORF1p (1:1000; Novus Biologicals, USA), and ORF2p (1:1000; Rockland, USA). The membranes were incubated overnight at 4 °C. Next day, membranes were washed three times with TBST (15 minutes per time) at room temperature to remove nonspecifically bound antibodies. Subsequently, membranes were incubated for 1 hour at room temperature with the goat anti-rabbit IgG HRP-conjugated secondary antibody (1:5000; Affinity, USA). Membranes were washed and signals were detected using the Tanon-5500 chemiluminescent imaging system (Tanon, China). Finally, band intensities were quantified using ImageJ software and the ORF1p and ORF2p chemiluminescent intensity was normalized to the loading control (β-Actin). All bands to be compared were run on the same gel and quantified on the same image.

**6 Quantification and Statistical Analysis**

Statistical analyses were performed by SPSS Statistics (v25.0; IBM, USA). First, the Shapiro-Wilk test was used to assess the normality of the data, confirming that all datasets followed a normal distribution. For comparisons between two groups, an unpaired Student's t-test was applied. One-way analysis of variance (ANOVA) with Tukey's multiple comparison was used to for multiple group comparisons. Linear correlations were evaluated using Pearson's correlation coefficient (*r*). To investigate the relationship of L1 content and protein with age, nonlinear regression analysis was employed, with *R^2^* as a statistic to assess the goodness-of-fit of the curve. The nature of sample analyzed in each experiment are listed in the figure legends. No outliers were excluded from analysis. Statistical significance was defined as **p* < 0.05, ***p* < 0.01, and ****p* < 0.001. All figures were prepared by GraphPad Prism (v9.0; GraphPad Software, USA).

**References**

Burns, K. H. (2020). Our Conflict with Transposable Elements and Its Implications for Human Disease. *Annu Rev Pathol*, *15*, 51-70. <https://doi.org/10.1146/annurev-pathmechdis-012419-032633>

Della Valle, F., Reddy, P., Yamamoto, M., Liu, P., Saera-Vila, A., Bensaddek, D.,…Orlando, V. (2022). LINE-1 RNA causes heterochromatin erosion and is a target for amelioration of senescent phenotypes in progeroid syndromes. *Sci Transl Med*, *14*(657), eabl6057. <https://doi.org/10.1126/scitranslmed.abl6057>

Dou, Z., Ghosh, K., Vizioli, M. G., Zhu, J., Sen, P., Wangensteen, K. J.,…Berger, S. L. (2017). Cytoplasmic chromatin triggers inflammation in senescence and cancer. *Nature*, *550*(7676), 402-406. <https://doi.org/10.1038/nature24050>

Guo, X., Li, J., Qi, Y., Chen, J., Jiang, M., Zhu, L.,…Wang, X. (2024). Telomere length and micronuclei trajectories in APP/PS1 mouse model of Alzheimer's disease: Correlating with cognitive impairment and brain amyloidosis in a sexually dimorphic manner. *Aging Cell*, *23*(5), e14121. <https://doi.org/10.1111/acel.14121>

Holtze, S., Gorshkova, E., Braude, S., Cellerino, A., Dammann, P., Hildebrandt, T. B.,…Sahm, A. (2021). Alternative Animal Models of Aging Research. *Front Mol Biosci*, *8*, 660959. <https://doi.org/10.3389/fmolb.2021.660959>

Jankowsky, J. L., Slunt, H. H., Ratovitski, T., Jenkins, N. A., Copeland, N. G., & Borchelt, D. R. (2001). Co-expression of multiple transgenes in mouse CNS: a comparison of strategies. *Biomol Eng*, *17*(6), 157-165. <https://doi.org/10.1016/s1389-0344(01)00067-3>

McKerrow, W., Wang, X., Mendez-Dorantes, C., Mita, P., Cao, S., Grivainis, M.,…Fenyö, D. (2022). LINE-1 expression in cancer correlates with p53 mutation, copy number alteration, and S phase checkpoint. *Proc Natl Acad Sci U S A*, *119*(8). <https://doi.org/10.1073/pnas.2115999119>

Simon, M., Van Meter, M., Ablaeva, J., Ke, Z., Gonzalez, R. S., Taguchi, T.,…Gorbunova, V. (2019). LINE1 Derepression in Aged Wild-Type and SIRT6-Deficient Mice Drives Inflammation. *Cell Metab*, *29*(4), 871-885.e875. <https://doi.org/10.1016/j.cmet.2019.02.014>
